# Criteria for priority setting of HIV/AIDS interventions in Thailand: a discrete choice experiment

**DOI:** 10.1186/1472-6963-10-197

**Published:** 2010-07-07

**Authors:** Sitaporn Youngkong, Rob Baltussen, Sripen Tantivess, Xander Koolman, Yot Teerawattananon

**Affiliations:** 1 Nijmegen International Center for Health Systems Research and Education (NICHE), Department of Primary and Community Care, Radboud University Nijmegen Medical Center, Nijmegen, The Netherlands; 2 Health Intervention and Technology Assessment Program (HITAP), Ministry of Public Health, Nonthaburi, Thailand; 3 International Health Policy Program (IHPP), Ministry of Public Health, Nonthaburi, Thailand; 4 Institute of Health Policy and Management, Erasmus University, Rotterdam, The Netherlands

## Abstract

**Background:**

Although a sizeable budget is available for HIV/AIDS control in Thailand, there will never be enough resources to implement every programme for all target groups at full scale. As such, there is a need to prioritize HIV/AIDS programmes. However, as of yet, there is no evidence on the criteria that should guide the priority setting of HIV/AIDS programmes in Thailand, including their relative importance. Also, it is not clear whether different stakeholders share similar preferences.

**Methods:**

Criteria for priority setting of HIV/AIDS interventions in Thailand were identified in group discussions with policy makers, people living with HIV/AIDS (PLWHA), and community members (i.e. village health volunteers (VHVs)). On the basis of these, discrete choice experiments were designed and administered among 28 policy makers, 74 PLWHA, and 50 VHVs.

**Results:**

In order of importance, policy makers expressed a preference for interventions that are highly effective, that are preventive of nature (as compared to care and treatment), that are based on strong scientific evidence, that target high risk groups (as compared to teenagers, adults, or children), and that target both genders (rather than only men or women). PLWHA and VHVs had similar preferences but the former group expressed a strong preference for care and treatment for AIDS patients.

**Conclusions:**

The study has identified criteria for priority setting of HIV/AIDS interventions in Thailand, and revealed that different stakeholders have different preferences vis-à-vis these criteria. This could be used for a broad ranking of interventions, and as such as a basis for more detailed priority setting, taking into account also qualitative criteria.

## Background

While the number of new HIV positive cases in Thailand decreases [1-3], HIV/AIDS continues to take a large toll in the country with 610,000 prevalent cases and approximately 30,000 deaths in 2007 [2]. A wide array of HIV/AIDS control programmes has been implemented to confront the epidemic since the first wave of infections in the mid-1980s [1,4]. Thailand's current national plan for HIV/AIDS prevention and alleviation, 2007-2011 [3] aims to: (i) integrate AIDS prevention, care, treatment, and impact reduction implementation into service provision at all levels; (ii) strengthen community's education about AIDS; (iii) enhance capacity of local administration in taking responsibility on local HIV/AIDS interventions; and (iv) prevent HIV transmission among children in schools and high-risk population groups. To date, the Thai government provides universal coverage for antiretroviral medication to all eligible people living with HIV/AIDS (PLWHA) [5]. However, HIV-related absenteeism and the need of informal care (e.g. care givers) have notable effects on individual PLWHA's economic burden [6].

Although a sizeable budget for implementation of this plan is available - approximately US$193 million in 2008 derived from both the Thai government and the International Monetary Fund [3] - there will never be enough resources to implement every programme for all target groups at full scale. As such, there is a need to prioritize HIV/AIDS programmes within the available budget, and to decide on which programmes will receive funding and which programmes will not.

A number of criteria can guide this priority setting process [7]. First, cost-effectiveness, or efficiency, aims to maximize population health given a certain budget. A limited number of cost-effectiveness analyses have been performed in Thailand [8], including (i) routine offer of HIV counseling and testing [9]; (ii) donated blood screening by nucleic acid testing [10]; (iii) HIV vaccination [11], and (iv) the prevention programme of mother-to-child transmission of HIV [12]. Secondly, equity or fairness, aims to minimize differences in health among population groups, with special reference to the severely ill, disadvantaged, or vulnerable populations [13]. Thirdly, the Thai government may hold preferences to target specific population groups, because they are more deserving of health care than others. The HIV/AIDS epidemics in Thailand concerns various population groups, including high-risk groups such as male homosexuals, intravenous drug users, and low-risk groups as the general population including teenagers, adults, and elderly [4,14]. In addition, a wide range of other medical (e.g. preference for acute care in life threatening situation) and non-medical criteria (e.g. preferences for programmes with desirable social consequences) may play a role in priority setting process [7,14-17].

It is clear that relying on a single criterion, e.g. efficiency, falls short to capture the important moral and ethical notions, and is unlikely to be acceptable for most policy makers [18-21] including those in Thailand [22]. The challenge for policy makers in Thailand is then to find the right balance between the various criteria. The trade-off a country like Thailand makes between e.g. efficiency and equity criteria can have important implications, e.g. adopting severity of disease rather than cost-effectiveness as guiding principle in the selection of HIV/AIDS interventions - and thus choosing treatment rather than prevention-centered strategies - could lead to a large number of extra infections in Thailand [14].

However, as of yet, there is no evidence on the criteria that should guide the priority setting of HIV/AIDS programmes in Thailand, including their relative importance. Also, it is not clear whether different stakeholders share similar preferences. It is against this background that this paper elicits preferences on the relative importance of criteria for priority setting of HIV/AIDS programmes in Thailand from policy makers, PLWHA and community members. The study uses discrete choice experiments (DCE) to elicit explicit preferences in HIV/AIDS area, and is the first study to do so in this area. The technique allows the assessment of the relative importance of different criteria that influence choice, in this case the priority setting of health interventions in HIV/AIDS control. The technique has shown promising results in a number of other disease areas in low-income settings [23-25]. The present study can hence be interpreted as exploratory, to test the feasibility of the approach, and have a first impression of its findings.

## Methods

### Discrete choice experiments

Discrete choice experiments are a quantitative methodology for evaluating the relative importance of the different product attributes that influence consumer choice behavior [26]. In such experiments, respondents are asked to make choices between hypothetical alternative goods or services.

We employed DCE to determine the relative importance of criteria for priority setting, according to various stakeholders. In a DCE, respondents choose their preferred option from sets of hypothetical scenarios, each consisting of a bundle of criteria that describe the scenario in question. The criteria are constant in each scenario, but the levels that describe each criterion may vary across scenarios. Analysis of the options chosen by respondents in each scenario reveals the extent to which each criterion is important to the decision at hand [27,28]. Running a DCE involves selection of participants, identification of criteria through group discussion, DCE design and administration of the DCE survey. These are discussed in turn.

### Participants

In this study, we chose to explore the views of policy makers in comparison with two other groups of stakeholders, i.e. PLWHA, and community members represented by village health volunteers (VHVs).

The policy makers were represented by 28 national - and province level decision makers strongly involved in health resource allocation decisions in Thailand specifically on HIV/AIDS. As a first step in the selection process, members of the National AIDS Committee were asked to participate. As a second step, they were asked to nominate other decision-makers meeting the above criterion. A total of 30 decision makers were invited, and 28 agreed to participate in the study. They were predominantly male (71.4%), and all being higher educated (bachelor degree or more) (Table 1).

**Table 1:** General characteristic of respondents

	Perspective					
	
	Policy makers		People living with HIV/AIDS		Village Health Volunteers	
	(n = 28)		(n = 74)		(n = 50)	
**Age (years)**						
mean (SD)	47.4 (6.9)		33.1 (5.5)		47.6 (9.0)	
**Gender**						
male	20	(71.4%)	28	(38.9%)	6	(12.0%)
female	8	(28.6%)	44	(61.1%)	44	(88.0%)
missing	-		2		-	
**Education**						
lower than bachelor	-		44	(61.1%)	40	(80.0%)
bachelor degree	4	(14.8%)	27	(37.5%)	10	(20.0%)
master degree	16	(59.3%)	1	(1.4%)	-	
doctoral degree	7	(25.9%)	-		-	
missing	1		2		-	
**Occupation**						
government officer	27	(100.0%)	1	(1.4%)	4	(8.0%)
private company employee	-		4	(5.6%)	3	(6.0%)
agriculturists	-		5	(7.0%)	2	(4.0%)
housewives	-		-		30	(60.0%)
freelancers/self-employee	-		54	(76%)	3	(6.0%)
others	-		7	(9.8%)	8	(16.0%)
missing	1		3		-	

The PLWHA were all members of the Thai network for people living with HIV/AIDS, representing PLWHA groups at the province and regional level in Thailand. In a regular network-meeting, we invited the members to participate in the present study. In total, 74 out of 85 invited PLWHA agreed to participate. They were predominantly female (61.1%) with a minority being higher educated.

The community members were represented by VHVs - these are community members who have been trained by public health providers in order to provide basic health care delivery including first aid and necessary health information to members of the village they reside in. In the selection process, we invited 100 VHVs in a semi-urban district of Samutprakan province, and out of these, 50 agreed to participate. They were predominantly female (88%), with a minority being higher educated.

### Identification of criteria and criteria levels

To define the criteria in DCE, group discussions were organized with each group of stakeholders including six representatives of that group. As an initial step, two HIV/AIDS interventions were presented. Then participants were asked to decide which intervention should be funded and reasons for the choices were discussed. The discussion was then broadened to discuss general reasons, or criteria, to fund HIV/AIDS interventions, and finally agreement was reached on a comprehensive set of criteria. Resulting criteria and associated levels from the three group discussions were compared. The final selection of criteria and levels included those that were identified by two or more discussion groups. This resulted in identification of one criterion at four levels, two criteria at three levels, and two criteria at two levels (Table 2).

**Table 2:** Attributes and levels

Attributes	Levels	Level coding	Definition
Target group	Children (Child)	Child	0 - 12 years old
	Teenagers	Teen	13 - 20 years old
	High risk adults	HiRisk	≥ 21 years old with high risk behavior e.g. sex workers,
			men who have sex with men, injected drug users,
			pregnant women, etc.
	All adults	Adults	≥ 21 years old without any specification
Gender of	Male	Male	aiming to male population
target group	Female	Female	aiming to female population
	Both genders	BothGen	not specify gender of target group
Type of	Treatment and care of patients	HIV	aiming to treat HIV infected people (CD4 ≥ 200) and
intervention	with HIV (not AIDS)		reduce HIV transmission
	Treatment and care of patients	AIDS	aiming to treat AIDS patients (CD4 < 200)
	with AIDS		
	Preventing HIV	Prevent	aiming to prevent general publics from HIV infection
Effectiveness	Low effective	LoEff	less than 50% of participants benefit
	High effective	HiEff	more than 50% of participants benefit
Quality of evidence	Weak evidence	Weak	no evidence but observation and/or expert opinions
on effectiveness	Strong evidence	Strong	evidence from domestic and/or international literatures

### DCE design

The DCE was designed on the following principles. To avoid information overload from a full factorial of 144 possible scenarios based on identified criteria and levels (4^1 ^× 3^2 ^× 2^2^), a limited number is chosen on the basis of a fractional experimental designs catalogue produced by Hahn and Shapiro [29]. The catalogue includes a number of orthogonal designs, both full factorial and fractional factorial ones, with differing numbers of attributes at differing numbers of levels. The fractional factorial design - fitting the number of identified criteria and levels - included a subset of 16 scenarios (representing an orthogonal array and minimizing multicollinearity), to allow the estimation of all main effects. Each of these 16 scenarios was paired by fold-over technique. A two-scenario with non-labeled experimental design was employed for each choice set. The plausibility of each scenario was evaluated with experts, policy makers, and in a pilot study with VHVs. The DCE questions are presented in additional file 1.

### DCE survey

The DCE survey was administrated to policy makers through face-to-face interview and to the other groups by self-administered questionnaires. For the latter groups, group meetings were organized to clarify the aims of the DCE survey and the questionnaire. At completion of the questionnaire, participants were asked to simply rank order the criteria included in the DCE on the basis of their importance in priority setting of HIV/AIDS interventions. To standardize and maintain quality of the data collection, the group discussion and interviews were conducted only by the first author.

### Data analysis

Regression coefficients, average marginal effects, and relative contributions were estimated from the response data by the statistical software program STATA 10.0. Regression coefficients indicate the sign of the effect of a variable on the probability of selection of an intervention. Since the response data is a dichotomous outcome - '1' is coded for being chosen, with '0' is coded for not being chosen - and dummy coding was used to transform the attribute levels into L-1 dummy variables in which each dummy is set equal to 1 when the qualitative level is present and set equal to 0 if it is not.

Binary logistic regression models were used to analyze the data, with the following description,(1)

where P is the probability of an intervention being selected by the respondents, β_0 _is the constant term, β_i _(i = 1-9) are the coefficients of the model indicating the probability of selection relative to the reference criterion level, and ε is the unobservable error term. To control for differences in attractiveness of DCE scenarios, dummies were added for scenarios to equation (1).

Marginal effects reflect the change in the probability of selection of an intervention. These were computed by taking the average difference in predicted probability of P with and without the variable, while holding the distribution of the other variables at their sample value, and then taking the sample mean of these differences.

The relative contributions were calculated to signify the contribution of one criterion to the variation in preferences explained by the regression model and therefore describe the relative importance of the various criteria in the choice of interventions. This relative importance depends on the variation in the levels that are chosen for each of the attributes. Variation explained by the model is based on Efron R^2^[30],(2)

where y_i _indicates the observed choice and  indicates the predicted probability that choice equal to 1. The relative contributions are calculated by computing Efron's R^2^of the above model minus Efron's R^2^of the model where the criterion is held constant at its sample mean. This procedure shows the contribution of criteria irrespective of the number of levels they have.

To test whether the decision makings on choice selections vary between perspectives, the likelihood ratio test was analyzed. At the end, as a validity check, the resulting rank ordering of attributes derived from DCE exercise was compared (presented by the relative contributions) to those derived from a simple rank ordering.

### Research ethics

This study was approved by Institute for the Development of Human Research Protections (IHRP), Ministry of Public Health, Thailand. All participants provided their written informed consent for the discussion and the interview.

## Results

Table 3 shows the results of logistic regression analysis and marginal effects calculation from the DCE response data, for each group of stakeholders. First, policy makers expressed a preference for highly effective interventions compared to those with low effectiveness, as indicated by the marginal effects. These show that the former interventions have a 38.5% higher probability of being selected than the latter. The next important criterion is intervention type, and policy makers expressed a preference for preventive interventions, followed by treatment of and care for HIV-infected people, and treatment of and care for AIDS-patients. The marginal effects show that preventive interventions have a 33% higher probability to be selected than the latter. Next, policy makers expressed a preference for interventions with strong evidence on intervention effectiveness compared to weak evidence. Also, policy makers preferred interventions that target high risk groups, followed by teenagers, adults, and children. Policy makers revealed a weak preference for gender of target group, with a priority to target both genders, followed by males and then females.

**Table 3:** Discrete choice model results and marginal effects by perspective

		Perspectives											
		
		Policy makers				People living with HIV/AIDS				Village Health Volunteers			
**Criteria**	**Levels**	***Coefficient*** (95% CI)		***(p-value)***	***Marginal effect***	***Coefficient*** (95% CI)		***(p-value)***	***Marginal effect***	***Coefficient*** (95% CI)		***(p-value)***	***Marginal effect***
Target group	Child												
	Teen	1.049*		(0.001)	0.183	0.135		(0.385)	0.030	0.830*		(0.000)	0.181
		(0.445, 1.654)				(-0.169, 0.440)				(0.464, 1.196)			
	HiRrisk	1.153*		(0.001)	0.199	0.022		(0.900)	0.005	0.314		(0.142)	0.069
		(0.502, 1.803)				(-0.323, 0.368)				(-0.105, 0.734)			
	Adults	0.023		(0.926)	0.004	-0.279		(0.065)	-0.061	-0.249		(0.176)	-0.054
		(-0.470, 0.517)				(-0.575, 0.017)				(-0.609, 0.112)			
Gender of target group	Male												
	Female	-0.256		(0.321)	-0.043	0.082		(0.544)	0.018	0.196		(0.229)	0.043
		(-0.762, 0.250)				(-0.184, 0.348)				(-0.123, 0.514)			
	BothGen	0.266		(0.189)	0.045	1.132*		(0.000)	0.255	0.724*		(0.000)	0.161
		(-0.131, 0.663)				(0.911, 1.354)				(0.458, 0.990)			
Type of intervention	HIV												
	AIDS	-0.493*		(0.019)	-0.088	1.091*		(0.000)	0.245	-0.476*		(0.001)	-0.105
		(-0.904, -0.081)				(0.869, 1.313)				(-0.744, -0.208)			
	Prevent	1.967*		(0.000)	0.333	0.212		(0.116)	0.047	0.246		(0.137)	0.054
		(1.450, 2.485)				(-0.052, 0.476)				(-0.078, 0.569)			
Effectiveness	LoEff												
	HiEff	1.983*		(0.000)	0.385	0.627*		(0.000)	0.140	1.185*		(0.000)	0.275
		(1.643, 2.323)				(0.454, 0.800)				(0.973, 1.395)			
Quality of evidence	Weak												
on effectiveness	Strong	1.310*		(0.000)	0.237	0.356*		(0.000)	0.079	0.349*		(0.001)	0.077
		(0.976, 1.645)				(0.183, 0.528)				(0.139, 0.560)			
Log likelihood		-424.4532				-1434.3323				-963.3818			
Pseudo R^2^		0.2747				0.0992				0.0984			
Hosmer-Lemeshow chi-square (p-value)		1.36 (0.995)				2.79 (0.947)				1.87 (0.985)			

*Significant variables (p < 0.05)

Likelihood ratio test (p < 0.000)

Second, PLWHA had similar preferences as policy makers with some exceptions. Most notably, PLWHA expressed a strong preference for treatment or care for AIDS patients, and the probability of selection of these interventions is 24.5% higher than treatment and care for HIV-infectious people. Moreover, they expressed a strong preference for targeting both genders rather than one gender only. Third, VHVs preferences cohered largely with that of policy makers.

The different models for policy makers, PLWHA and VHVs demonstrate a good fit as indicated by the pseudo R^2^, and Hosmer-Lemeshow chi-square. The likelihood ratio test presents that the preferences on the criteria of each group of stakeholders are significantly different.

The contribution R^2 ^indicates the overall importance of criteria (Table 4). Policy makers considered intervention effectiveness as the most important criterion, followed by intervention type, quality of evidence, target group, and gender of target group. PLWHA considered gender of target group as most important criterion, followed by intervention type, intervention effectiveness, quality of evidence, and target group. VHVs considered intervention effectiveness as most important, followed by target group, gender of target group, type of intervention, and quality of evidence. Table 4 also shows the results of the simple rank ordering of criteria, and it reveals large overlaps for the policy makers, but less so for PLWHA and VHVs.

**Table 4:** Rank ordering of criteria in simple ranking and DCE exercise

	Perspective					
	
	Policy makers		People living with HIV/AIDS		Village Health Volunteers	
**Simple ranking**						

Rank 1	Effectiveness		Target group		Target group	
Rank 2	Target group		Effectiveness		Gender of target group	
Rank 3	Type of intervention		Quality of evidence		Type of intervention	
Rank 4	Quality of evidence		Type of intervention		Effectiveness	
Rank 5	Gender of target group		Gender of target group		Quality of evidence	

**DCE**		R^2^		R^2^		R^2^

Rank 1	Effectiveness	0.152	Gender of target group	0.058	Effectiveness	0.075
Rank 2	Type of intervention	0.091	Type of intervention	0.042	Target group	0.017
Rank 3	Quality of evidence	0.053	Effectiveness	0.019	Gender of target group	0.016
Rank 4	Target group	0.015	Quality of evidence	0.008	Type of intervention	0.013
Rank 5	Gender of target group	0.014	Target group	0.003	Quality of evidence	0.006

DCE, discrete choice experiments; R^2^, contribution R^2^

## Discussion and Conclusions

The study has identified criteria for priority setting of HIV/AIDS interventions in Thailand using perspective of policy makers, PLWHA, and VHVs, and revealed that different stakeholders have different preferences vis-à-vis these criteria. A number of observations can be made.

First, the findings show that policy makers give priority to preventing HIV interventions, and targeting high risk populations. This is in line with the Thai national policy on priority setting of HIV interventions, which focuses on prevention among people who may be the most at risk of transmitting HIV [3]. Yet, although policy makers may put higher priority on HIV prevention programs, it is obvious that therapy cannot be neglected [3,14,19]. The Thai national HIV/AIDS plan emphasizes integrating HIV and AIDS prevention and treatment programs [3,31]. The emphasis on intervention effectiveness and related quality of evidence confirms the importance that is attributed to evidence-based medicine in Thailand [8].

Secondly, the study reveals large similarities in the preferences for criteria for HIV/AIDS interventions between policy makers and VHVs. This may indicate that the preferences of community members (based on the sample used) are well reflected through decisions made by policy makers. This study also highlights the differences in preferences between PLWHA and the other stakeholder groups. The preferences of the former for care and treatment may reflect self-interests, whereas the preferences of the latter may reflect preferences for the society at large.

Thirdly, our findings show overlap between the ranking of criteria resulting from DCE and as obtained from simple ranking for policy makers, but less so for other stakeholders. This may indicate validity and hence usefulness of DCE for (well-educated) policy makers, but possibly less so for other (less-educated) stakeholders.

Recently, a number of empirical priority setting studies have included the views of different stakeholders, such as patients and community members, besides those of policy makers [32]. Inclusion of different perspectives is important, to enhance the legitimacy of the priority setting process, as has been acknowledged in the Accountability for Reasonableness framework [33]. The present study is a first step to integrate different views, by documenting differences and similarities. The study did not aim to reach consensus by the different stakeholders, and it is not sure which methodology could be used to accommodate this. A challenge here is to avoid dominance by one group stakeholder (e.g. policy makers) over another (e.g. community members).

The DCE in this study only includes the criteria that were found to overlap from the focus group discussions. The rationale for doing so was to accommodate comparability of study findings (so to include identical criteria in DCE for the various stakeholders) on the one hand, while maintaining the number of criteria to a manageable number (thus not including all possible criteria that were put forward by any discussion group) on the other hand. However, this choice may have led to the omission of important criteria for some groups of stakeholders, and may have reduced the validity of study findings. Next studies should seek to strike a balance between comparability and validity.

Our study findings are based on small sample sizes (ranging from 28 for policy makers, to 74 for PLWHA), and should therefore be interpreted with caution. This also indicates the explorative character of our study. A proper sample size calculation is difficult in the absence a prior information on the variances on the responses - we based our sample sizes on previous similar studies, e.g. in Ghana [23] and Nepal [24] that also included a limited number of respondents.

Intervention utility can be calculated by assuming a main effects additive utility model on the basis of a linear combination of the weights of each level of all criteria [28]. This utility can then be compared to costs, to derivate a cost-utility estimate. Subsequently, interventions can then be rank ordered on the basis of these cost-utility estimates, and this rank ordering reflects the overall intervention attractiveness. A rank ordering of HIV/AIDS interventions on the basis of cost-utility information can be used to inform policy decision making, and is topic for further research. This is conceptually more consistent approach than considering cost-effectiveness as a separate criterion, as applied in other similar studies [23,25]. However, cost-effectiveness as a separate criterion also has much appeal to policy makers, and is not clear which approach is best in supporting decision in real-life.

The present study findings, and associated rank ordering of HIV/AIDS interventions, can be considered as general principles to prioritization of HIV/AIDS interventions in Thailand. Since the DCE design only involves a set of criteria amendable to quantification, it ignores a range of non-quantifiable considerations - e.g. ethical, political, and social concerns [34,35]. As such, any rank ordering of intervention can be indicative only, and should never be interpreted in a mathematical manner. In this respect, a broad clustering or typology of interventions that are probable 'good candidates for implementation', 'not good candidates for implementation', and 'in-between' is perhaps a good way to present results to policy makers. Such a broad typology is then a starting point for a more detailed priority setting process, in which policy makers can still deviate from the broad recommendations. A deliberative process is able to include the non-quantitative criteria and can encourage participatory approaches with a variety of stakeholders and interests [36,37].

This exploratory study has shown the feasibility of eliciting explicit preferences on the criteria for prioritization of HIV/AIDS interventions in Thailand. Further studies should refine methodological aspects, and interpret the findings in terms of the prioritization of interventions.

## Competing interests

The authors declare that they have no competing interests.

## Authors' contributions

SY and RB conceptualized the study. SY carried out the data collection, and drafted the manuscript. RB, ST and YT participated in the study design, made substantial contributions to the data interpretation and writing of the paper. XK participated in data analysis. All authors read and approved the final manuscript.

## Pre-publication history

The pre-publication history for this paper can be accessed here:

http://www.biomedcentral.com/1472-6963/10/197/prepub

## Supplementary Material

Additional file 1 **The DCE questionnaire**. The questionnaire presents the DCE questions and explanatory notes used in the survey (10 pages) Click here for file
