# Supplementary material for: Criteria for priority setting of HIV/AIDS interventions in Thailand: a discrete choice experiment
Source: BMC Health Serv Res. 2010 Jul 7;10:197. doi: 10.1186/1472-6963-10-197 (PMC2912896; doi:10.1186/1472-6963-10-197)
Supplement: Additional file 1 — The DCE questionnaire. The questionnaire presents the DCE questions and explanatory notes used in the survey (10 pages) [file 1472-6963-10-197-S1.DOC]

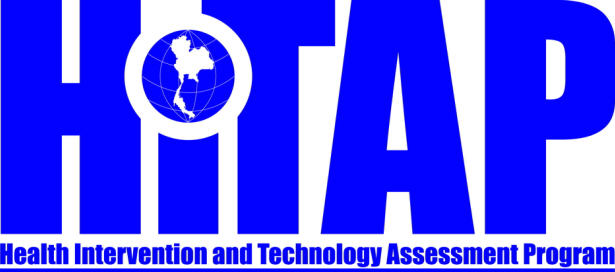

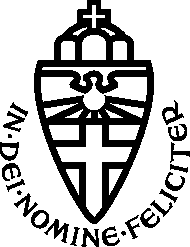


**Developing of Guidelines**

**for Priority Setting of HIV/AIDS Interventions in Thailand**

**by**

**Health Intervention and Technology Assessment Program (HITAP)**

**Ministry of Public Health, Thailand**

**and**

**Nijmegen International Center for Health Systems Research and Education (NICHE), Radboud University Nijmegen Medical Centre,**

**The Netherlands**

**Discrete Choice Experiments**

Suppose that you were a Thai health policy maker faced with priority setting decisions on how to allocate scarce budgets. Given that only one of the two options below can receive funding, which one would you choose?

| **Choice set 1** |  |  | **A** |  | |  |  | **B** |  | | |
| --- | --- | --- | --- | --- | --- | --- | --- | --- | --- | --- | --- |
| Target group |  | children | | | |  | teenagers | | | |  |
| Gender of target group |  | female | | | |  | male | | | |  |
| Type of intervention |  | preventing HIV | | | |  | treatment of patients with HIV | | | |  |
| Effectiveness |  | high effective | | | |  | low effective | | | |  |
| Quality of evidence on effectiveness |  | strong evidence | | | |  | weak evidence | | | |  |
|  |  |  |  | |  |  |  |  | |  | |
| Which one would you choose? Please tick a box |  |  |  | |  |  |  |  | |  | |
|  |  |  |  | |  |  |  |  | |  | |

*Explanatory note*

Option A An intervention aims to prevent girls who are 0-12 years old from HIV infection. There is evidence from domestic or international literature presenting that more than 50% of participants can protect themselves from HIV infection.

Option B An intervention aims to treat HIV infected men who are 13-20 years old. There is no evidence to support the effectiveness of this intervention; however, experts believe that less than 50% of participants can be treated or can reduce HIV transmission.

| **Choice set 2** |  |  | **A** |  | |  |  | **B** |  | | |
| --- | --- | --- | --- | --- | --- | --- | --- | --- | --- | --- | --- |
| Target group |  | teenagers | | | |  | high risk adults | | | |  |
| Gender of target group |  | female | | | |  | male | | | |  |
| Type of intervention |  | treatment of patients with HIV | | | |  | treatment of patients with AIDS | | | |  |
| Effectiveness |  | low effective | | | |  | high effective | | | |  |
| Quality of evidence on effectiveness |  | weak evidence | | | |  | strong evidence | | | |  |
|  |  |  |  | |  |  |  |  | |  | |
| Which one would you choose? Please tick a box |  |  |  | |  |  |  |  | |  | |
|  |  |  |  | |  |  |  |  | |  | |

*Explanatory note*

Option A An intervention aims to treat HIV infected women who are 13-20 years old. There is no evidence to support the effectiveness of this intervention; however, experts believe that less than 50% of participants can be treated or can reduce HIV transmission.

Option B An intervention aims to treat male AIDS patients who are ≥21 years old and have highly risky behavior such as injecting drug users and homosexuals. There is evidence from domestic or international literature presenting that more than 50% of participants can be treated or can prolong their lives.

| **Choice set 3** |  |  | **A** |  | |  |  | **B** |  | | |
| --- | --- | --- | --- | --- | --- | --- | --- | --- | --- | --- | --- |
| Target group |  | high risk adults | | | |  | all adults | | | |  |
| Gender of target group |  | female | | | |  | male | | | |  |
| Type of intervention |  | treatment of patients with AIDS | | | |  | Preventing HIV | | | |  |
| Effectiveness |  | high effective | | | |  | low effective | | | |  |
| Quality of evidence on effectiveness |  | weak evidence | | | |  | strong evidence | | | |  |
|  |  |  |  | |  |  |  |  | |  | |
| Which one would you choose? Please tick a box |  |  |  | |  |  |  |  | |  | |
|  |  |  |  | |  |  |  |  | |  | |

*Explanatory note*

Option A An intervention aims to treat female AIDS patients who are ≥21 years old and have highly risky behavior such as female sex workers. There is no evidence to support the effectiveness of this intervention; however, experts believe that more than 50% of participants can be treated or can prolong their lives.

Option B An intervention aims to prevent men who are ≥21 years old from HIV infection. There is evidence from domestic or international literature presenting that less than 50% of participants can protect themselves from HIV infection.

| **Choice set 4** |  |  | **A** |  | |  |  | **B** |  | | |
| --- | --- | --- | --- | --- | --- | --- | --- | --- | --- | --- | --- |
| Target group |  | all adults | | | |  | children | | | |  |
| Gender of target group |  | female | | | |  | male | | | |  |
| Type of intervention |  | treatment of patients with HIV | | | |  | treatment of patients with AIDS | | | |  |
| Effectiveness |  | low effective | | | |  | high effective | | | |  |
| Quality of evidence on effectiveness |  | strong evidence | | | |  | weak evidence | | | |  |
|  |  |  |  | |  |  |  |  | |  | |
| Which one would you choose? Please tick a box |  |  |  | |  |  |  |  | |  | |
|  |  |  |  | |  |  |  |  | |  | |

*Explanatory note*

Option A An intervention aims to treat HIV infected women who are ≥21 years old. There is evidence from domestic or international literature presenting that less than 50% of participants can be treated or can reduce HIV transmission.

Option B An intervention aims to treat male AIDS patients who are 0-12 years old. There is no evidence to support the effectiveness of this intervention; however, experts believe that more than 50% of participants can be treated or can prolong their lives.

| **Choice set 5** |  |  | **A** |  | |  |  | **B** |  | | |
| --- | --- | --- | --- | --- | --- | --- | --- | --- | --- | --- | --- |
| Target group |  | children | | | |  | teenagers | | | |  |
| Gender of target group |  | male | | | |  | both genders | | | |  |
| Type of intervention |  | treatment of patients with HIV | | | |  | treatment of patients with AIDS | | | |  |
| Effectiveness |  | low effective | | | |  | high effective | | | |  |
| Quality of evidence on effectiveness |  | strong evidence | | | |  | weak evidence | | | |  |
|  |  |  |  | |  |  |  |  | |  | |
| Which one would you choose? Please tick a box |  |  |  | |  |  |  |  | |  | |
|  |  |  |  | |  |  |  |  | |  | |

*Explanatory note*

Option A An intervention aims to treat HIV infected boys who are 0-12 years old. There is evidence from domestic or international literature presenting that less than 50% of participants can be treated of can reduce HIV transmission.

Option B An intervention aims to treat AIDS patients who are 13-20 years old. There is no evidence to support the effectiveness of this intervention; however, experts believe that more than 50% of participants can be treated or can prolong their lives.

| **Choice set 6** |  |  | **A** |  | |  |  | **B** |  | | |
| --- | --- | --- | --- | --- | --- | --- | --- | --- | --- | --- | --- |
| Target group |  | teenagers | | | |  | high risk adults | | | |  |
| Gender of target group |  | male | | | |  | both genders | | | |  |
| Type of intervention |  | preventing HIV | | | |  | treatment of patients with HIV | | | |  |
| Effectiveness |  | high effective | | | |  | low effective | | | |  |
| Quality of evidence on effectiveness |  | weak evidence | | | |  | strong evidence | | | |  |
|  |  |  |  | |  |  |  |  | |  | |
| Which one would you choose? Please tick a box |  |  |  | |  |  |  |  | |  | |
|  |  |  |  | |  |  |  |  | |  | |

*Explanatory note*

Option A An intervention aims to prevent men who are 13-20 years old from HIV infection. There is no evidence to support the effectiveness of this intervention; however, experts believe that more than 50% of participants can protect themselves from HIV infection.

Option B An intervention aims to treat HIV infected people who are ≥21 years old and have highly risky behavior such as female sex workers, injecting drug users, and homosexuals. There is evidence from domestic or international literature presenting that less than 50% of participants can be treated or can reduce HIV transmission.

| **Choice set 7** |  |  | **A** |  | |  |  | **B** |  | | |
| --- | --- | --- | --- | --- | --- | --- | --- | --- | --- | --- | --- |
| Target group |  | high risk adults | | | |  | all adults | | | |  |
| Gender of target group |  | male | | | |  | both genders | | | |  |
| Type of intervention |  | treatment of patients with HIV | | | |  | treatment of patients with AIDS | | | |  |
| Effectiveness |  | low effective | | | |  | high effective | | | |  |
| Quality of evidence on effectiveness |  | weak evidence | | | |  | strong evidence | | | |  |
|  |  |  |  | |  |  |  |  | |  | |
| Which one would you choose? Please tick a box |  |  |  | |  |  |  |  | |  | |
|  |  |  |  | |  |  |  |  | |  | |

*Explanatory note*

Option A An intervention aims to treat HIV infected men who are ≥21 years old and have highly risky behavior such as injecting drug users, and homosexuals. There is no evidence to support the effectiveness of this intervention; however, experts believe that less than 50% of participants can be treated or can reduce HIV transmission.

Option B An intervention aims to treat AIDS patients who are ≥21 years old. There is evidence from domestic or international literature presenting that more than 50% of participants can be treated or can prolong their lives.

| **Choice set 8** |  |  | **A** |  | |  |  | **B** |  | | |
| --- | --- | --- | --- | --- | --- | --- | --- | --- | --- | --- | --- |
| Target group |  | all adults | | | |  | children | | | |  |
| Gender of target group |  | male | | | |  | both genders | | | |  |
| Type of intervention |  | treatment of patients with AIDS | | | |  | preventing HIV | | | |  |
| Effectiveness |  | high effective | | | |  | low effective | | | |  |
| Quality of evidence on effectiveness |  | strong evidence | | | |  | weak evidence | | | |  |
|  |  |  |  | |  |  |  |  | |  | |
| Which one would you choose? Please tick a box |  |  |  | |  |  |  |  | |  | |
|  |  |  |  | |  |  |  |  | |  | |

*Explanatory note*

Option A An intervention aims to treat male AIDS patients who are ≥21 years old. There is evidence from domestic or international literature presenting that more than 50% of participants can be treated or can prolong their lives.

Option B An intervention aims to prevent children who are 0-12 years old from HIV infection. There is no evidence to support the effectiveness of this intervention; however, experts believe that less than 50% of participants can protect themselves from HIV infection.

| **Choice set 9** |  |  | **A** |  | |  |  | **B** |  | | |
| --- | --- | --- | --- | --- | --- | --- | --- | --- | --- | --- | --- |
| Target group |  | children | | | |  | teenagers | | | |  |
| Gender of target group |  | both genders | | | |  | female | | | |  |
| Type of intervention |  | treatment of patients with AIDS | | | |  | preventing HIV | | | |  |
| Effectiveness |  | low effective | | | |  | high effective | | | |  |
| Quality of evidence on effectiveness |  | weak evidence | | | |  | strong evidence | | | |  |
|  |  |  |  | |  |  |  |  | |  | |
| Which one would you choose? Please tick a box |  |  |  | |  |  |  |  | |  | |
|  |  |  |  | |  |  |  |  | |  | |

*Explanatory note*

Option A An intervention aims to treat AIDS patients who are 0-12 years old. There is no evidence to support the effectiveness of this intervention; however, experts believe that less than 50% of participants can be treated or can prolong their lives.

Option B An intervention aims to prevent women who are 13-20 years old from HIV infection. There is evidence from domestic or international literature presenting that more than 50% of participants can protect themselves from HIV infection.

| **Choice set 10** |  |  | **A** |  | |  |  | **B** |  | | |
| --- | --- | --- | --- | --- | --- | --- | --- | --- | --- | --- | --- |
| Target group |  | teenagers | | | |  | high risk adults | | | |  |
| Gender of target group |  | both genders | | | |  | female | | | |  |
| Type of intervention |  | treatment of patients with HIV | | | |  | treatment of patients with AIDS | | | |  |
| Effectiveness |  | high effective | | | |  | low effective | | | |  |
| Quality of evidence on effectiveness |  | strong evidence | | | |  | weak evidence | | | |  |
|  |  |  |  | |  |  |  |  | |  | |
| Which one would you choose? Please tick a box |  |  |  | |  |  |  |  | |  | |
|  |  |  |  | |  |  |  |  | |  | |

*Explanatory note*

Option A An intervention aims to treat HIV infected people who are 13-20 years old. There is evidence from domestic or international literature presenting that more than 50% of participants can be treated or can reduce HIV transmission.

Option B An intervention aims to treat female AIDS patients who are ≥21 years old and have highly risky behavior such as female sex workers. There is no evidence to support the effectiveness of this intervention; however, experts believe that less than 50% of participants can be treated or can prolong their lives.

| **Choice set 11** |  |  | **A** |  | |  |  | **B** |  | | |
| --- | --- | --- | --- | --- | --- | --- | --- | --- | --- | --- | --- |
| Target group |  | high risk adults | | | |  | all adults | | | |  |
| Gender of target group |  | both genders | | | |  | female | | | |  |
| Type of intervention |  | preventing HIV | | | |  | treatment of patients with HIV | | | |  |
| Effectiveness |  | low effective | | | |  | high effective | | | |  |
| Quality of evidence on effectiveness |  | strong evidence | | | |  | weak evidence | | | |  |
|  |  |  |  | |  |  |  |  | |  | |
| Which one would you choose? Please tick a box |  |  |  | |  |  |  |  | |  | |
|  |  |  |  | |  |  |  |  | |  | |

*Explanatory note*

Option A An intervention aims to prevent people who are ≥21 years old and have highly risky behavior such as female sex workers, injecting drug users, and homosexuals from HIV infection. There is evidence from domestic or international literature presenting that less than 50% of participants can protect themselves from HIV infection.

Option B An intervention aims to treat HIV infected women who are ≥21 years old. There is no evidence to support the effectiveness of this intervention; however, experts believe that more than 50% of participants can be treated or can reduce HIV transmission.

| **Choice set 12** |  |  | **A** |  | |  |  | **B** |  | | |
| --- | --- | --- | --- | --- | --- | --- | --- | --- | --- | --- | --- |
| Target group |  | all adults | | | |  | children | | | |  |
| Gender of target group |  | both genders | | | |  | female | | | |  |
| Type of intervention |  | treatment of patients with HIV | | | |  | treatment of patients with AIDS | | | |  |
| Effectiveness |  | high effective | | | |  | low effective | | | |  |
| Quality of evidence on effectiveness |  | weak evidence | | | |  | strong evidence | | | |  |
|  |  |  |  | |  |  |  |  | |  | |
| Which one would you choose? Please tick a box |  |  |  | |  |  |  |  | |  | |
|  |  |  |  | |  |  |  |  | |  | |

*Explanatory note*

Option A An intervention aims to treat HIV infected people who are ≥21 years old. There is no evidence to support the effectiveness of this intervention; however, experts believe that more than 50% of participants can be treated or can reduce HIV transmission.

Option B An intervention aims to treat female AIDS patients who are 0-12 years old. There is evidence from domestic or international literature presenting that less than 50% of participants can be treated or can prolong their lives.

| **Choice set 13** |  |  | **A** |  | |  |  | **B** |  | | |
| --- | --- | --- | --- | --- | --- | --- | --- | --- | --- | --- | --- |
| Target group |  | children | | | |  | teenagers | | | |  |
| Gender of target group |  | male | | | |  | both genders | | | |  |
| Type of intervention |  | treatment of patients with HIV | | | |  | treatment of patients with AIDS | | | |  |
| Effectiveness |  | high effective | | | |  | low effective | | | |  |
| Quality of evidence on effectiveness |  | weak evidence | | | |  | strong evidence | | | |  |
|  |  |  |  | |  |  |  |  | |  | |
| Which one would you choose? Please tick a box |  |  |  | |  |  |  |  | |  | |
|  |  |  |  | |  |  |  |  | |  | |

*Explanatory note*

Option A An intervention aims to treat HIV infected boys who are 0-12 years old. There is no evidence to support the effectiveness of this intervention; however, experts believe that more than 50% of participants can be treated or can reduce HIV transmission.

Option B An intervention aims to treat AIDS patients who are 13-20 years old. There is evidence from domestic or international literature presenting that less than 50% of participants can be treated or can prolong their lives.

| **Choice set 14** |  |  | **A** |  | |  |  | **B** |  | | |
| --- | --- | --- | --- | --- | --- | --- | --- | --- | --- | --- | --- |
| Target group |  | teenagers | | | |  | high risk adults | | | |  |
| Gender of target group |  | male | | | |  | both genders | | | |  |
| Type of intervention |  | treatment of patients with AIDS | | | |  | preventing HIV | | | |  |
| Effectiveness |  | low effective | | | |  | high effective | | | |  |
| Quality of evidence on effectiveness |  | strong evidence | | | |  | weak evidence | | | |  |
|  |  |  |  | |  |  |  |  | |  | |
| Which one would you choose? Please tick a box |  |  |  | |  |  |  |  | |  | |
|  |  |  |  | |  |  |  |  | |  | |

*Explanatory note*

Option A An intervention aims to treat male AIDS patients who are 13-20 years old. There is evidence from domestic or international literature presenting that less than 50% of participants can be treated or can prolong their lives.

Option B An intervention aims to prevent people who are ≥21 years old and have highly risky behavior such as female sex workers, injecting drug users, and homosexuals from HIV infection. There is no evidence to support the effectiveness of this intervention; however, experts believe that more than 50% of participants can protect themselves from HIV infection.

| **Choice set 15** |  |  | **A** |  | |  |  | **B** |  | | |
| --- | --- | --- | --- | --- | --- | --- | --- | --- | --- | --- | --- |
| Target group |  | high risk adults | | | |  | all adults | | | |  |
| Gender of target group |  | male | | | |  | both genders | | | |  |
| Type of intervention |  | treatment of patients with HIV | | | |  | treatment of patients with AIDS | | | |  |
| Effectiveness |  | high effective | | | |  | low effective | | | |  |
| Quality of evidence on effectiveness |  | strong evidence | | | |  | weak evidence | | | |  |
|  |  |  |  | |  |  |  |  | |  | |
| Which one would you choose? Please tick a box |  |  |  | |  |  |  |  | |  | |
|  |  |  |  | |  |  |  |  | |  | |

*Explanatory note*

Option A An intervention aims to treat HIV infected men who are ≥21 years old and have highly risky behavior such as injecting drug users, and homosexuals. There is evidence from domestic or international literature presenting that more than 50% of participants can be treated or can reduce HIV transmission.

Option B An intervention aims to treat AIDS patients who are ≥21 years old. There is no evidence to support the effectiveness of this intervention; however, experts believe that less than 50% of participants can be treated or can prolong their lives.

| **Choice set 16** |  |  | **A** |  | |  |  | **B** |  | | |
| --- | --- | --- | --- | --- | --- | --- | --- | --- | --- | --- | --- |
| Target group |  | all adults | | | |  | children | | | |  |
| Gender of target group |  | male | | | |  | both genders | | | |  |
| Type of intervention |  | preventing HIV | | | |  | treatment of patients with HIV | | | |  |
| Effectiveness |  | low effective | | | |  | high effective | | | |  |
| Quality of evidence on effectiveness |  | weak evidence | | | |  | strong evidence | | | |  |
|  |  |  |  | |  |  |  |  | |  | |
| Which one would you choose? Please tick a box |  |  |  | |  |  |  |  | |  | |
|  |  |  |  | |  |  |  |  | |  | |

*Explanatory note*

Option A An intervention aims to prevent men who are ≥21 years old from HIV infection. There is no evidence to support the effectiveness of this intervention; however, experts believe that less than 50% of participants can protect themselves from HIV infection.

Option B An intervention aims to treat HIV infected children who are 0-12 years old. There is evidence from domestic or international literature presenting that more than 50% of participants can be treated or can reduce HIV transmission.

**-- Thank you for your cooperation --**
